# The Long-Term Stability of Intracortical Microstimulation and the Foreign Body Response Are Layer Dependent

**DOI:** 10.3389/fnins.2022.908858

**Published:** 2022-06-13

**Authors:** Morgan E. Urdaneta, Nicolas G. Kunigk, Seth Currlin, Francisco Delgado, Shelley I. Fried, Kevin J. Otto

**Affiliations:** ^1^Department of Neuroscience, University of Florida, Gainesville, FL, United States; ^2^J. Crayton Pruitt Family Department of Biomedical Engineering, University of Florida, Gainesville, FL, United States; ^3^Department of Neurosurgery, Massachusetts General Hospital, Harvard Medical School, Boston, MA, United States; ^4^Boston Veterans Affairs Healthcare System, Boston, MA, United States; ^5^Department of Materials Science and Engineering, University of Florida, Gainesville, FL, United States; ^6^Department of Neurology, University of Florida, Gainesville, FL, United States; ^7^Department of Electrical and Computer Engineering, University of Florida, Gainesville, FL, United States

**Keywords:** neuroprostheses, cortex, brain computer interface, foreign body response (FBR), microelectrode array, glial scar, microglia

## Abstract

Intracortical microstimulation (ICMS) of the somatosensory cortex (S1) can restore sensory function in patients with paralysis. Studies assessing the stability of ICMS have reported heterogeneous responses across electrodes and over time, potentially hindering the implementation and translatability of these technologies. The foreign body response (FBR) and the encapsulating glial scar have been associated with a decay in chronic performance of implanted electrodes. Moreover, the morphology, intrinsic properties, and function of cells vary across cortical layers, each potentially affecting the sensitivity to ICMS as well as the degree of the FBR across cortical depth. However, layer-by-layer comparisons of the long-term stability of ICMS as well as the extent of the astrocytic glial scar change across cortical layers have not been well explored. Here, we implanted silicon microelectrodes with electrode sites spanning all the layers of S1 in rats. Using a behavioral paradigm, we obtained ICMS detection thresholds from all cortical layers for up to 40 weeks. Our results showed that the sensitivity and long-term performance of ICMS is indeed layer dependent. Overall, detection thresholds decreased during the first 7 weeks post-implantation (WPI). This was followed by a period in which thresholds remained stable or increased depending on the interfacing layer: thresholds in L1 and L6 exhibited the most consistent increases over time, while those in L4 and L5 remained the most stable. Furthermore, histological investigation of the tissue surrounding the electrode showed a biological response of microglia and macrophages which peaked at L1, while the area of the astrocytic glial scar peaked at L2/3. Interestingly, the biological response of these FBR markers is less exacerbated at L4 and L5, suggesting a potential link between the FBR and the long-term stability of ICMS. These findings suggest that interfacing depth can play an important role in the design of chronically stable implantable microelectrodes.

## Introduction

The performance of brain-machine interface (BMI) technologies can be improved by receiving sensory feedback through intracortical microstimulation (ICMS) (Romo et al., 1998; Flesher et al., 2016; Salas et al., 2018). Clinical work has shown that ICMS of somatosensory cortex (S1) can elicit touch percepts in patients with paralysis (Flesher et al., 2016). The use of implantable microelectrodes allows for the spatial selectivity (Urdaneta et al., 2017) necessary to elicit naturalistic touch percepts in specific areas of the arm (Salas et al., 2018) or individual fingers (Flesher et al., 2016). While this is encouraging, the widespread implementation of ICMS technologies for chronic use requires further understanding of the challenges inherently associated with implantable microelectrodes, such as the foreign body response (FBR) (Biran et al., 2005) and the long-term stability of ICMS.

Studies assessing the stability of ICMS have found heterogeneous responses over time (Rousche and Normann, 1999; Bartlett et al., 2005; Koivuniemi et al., 2011; Davis et al., 2012; Callier et al., 2015; Hughes et al., 2021). Some reports find an immediate rise in microstimulation detection thresholds (Davis et al., 2012); in contrast, others report an initial decrease in thresholds followed by a period of either stable (Callier et al., 2015; Hughes et al., 2021) or increasing thresholds (Rousche and Normann, 1999; Koivuniemi et al., 2011).

One potential contributing factor to these heterogeneous responses is the FBR (Biran et al., 2005; Salatino et al., 2017), which has been associated with a decay in chronic performance of implanted microelectrodes (Prasad and Sanchez, 2012; Barrese et al., 2013). The disruption of the blood-brain barrier and the continuous presence of the device (Kozai et al., 2012) leads to infiltration of bloodborne macrophages (Woolley et al., 2013) and activation of microglia (Eles et al., 2017). These cells release proinflammatory cytokines (Giulian et al., 1994) that lead to the activation of astrocytes and the formation of an encapsulating glial scar around the electrode (Salatino et al., 2017; Campbell and Wu, 2018) that can act as a barrier limiting the delivery of charge to nearby neurons (Mercanzini et al., 2009).

Another potential factor leading to diverse ICMS stability responses is the cortical depth of the electrode interface. Indeed, studies have shown that both the intensity of the FBR (McConnell et al., 2009; Woolley et al., 2013; Kozai et al., 2014; Nolta et al., 2015) as well as the sensitivity of neurons to ICMS is non-uniform across cortical depth (DeYoe et al., 2005; Tehovnik and Slocum, 2009; Urdaneta et al., 2021). Given that the cellular morphology (Gouwens et al., 2019) and cortical dynamics (Harris and Shepherd, 2015) of the cortex are layer specific, the interfacing layer of the electrode can play an important role in the degree of the FBR and the long-term stability of the ICMS. However, to our knowledge, these questions remained unexplored.

Here, we compare the response to ICMS over time as a function of cortical depth and show an association between FBR and ICMS sensitivity at different cortical layers. For this, we implanted a silicon microelectrode device with electrode sites spanning all layers of the S1 cortex in rat. We measured ICMS detection thresholds at each site (i.e., across cortical depth) up to 40 weeks post-implantation (WPI). Post-mortem we histologically examined the area of the astrocytic glial scar and the microglial response across cortical depth. Our results show that the stability of ICMS changes over time in a layer-specific manner. Moreover, the extent of the astrocytic glial scar and microglia was dependent on cortical depth. These findings provide insights into the role of cortical depth in the FBR and the long-term stability of ICMS delivered *via* intracortical microelectrodes.

## Results

**FIGURE 1:**
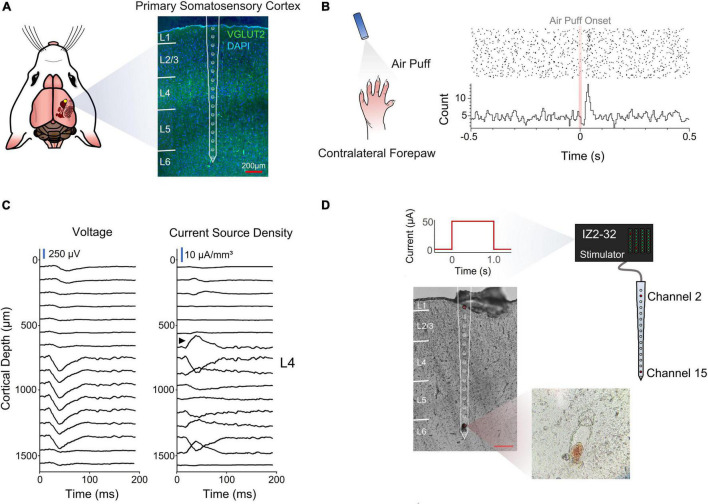
Electrode implantation and cortical depth assessment. **(A)** Coronal section of the implantation site stained for VGLUT2 and DAPI for cortical layer identification. **(B)** Perievent stimulus time histogram of an air puff onset to the contralateral forepaw. **(C)** Voltage traces and inverse current source density. Black arrow designates sink characteristic of thalamic input into layer 4. **(D)** Post-mortem depth assessment *via* electrolytic lesion of the tissue.

Rats were chronically implanted with a silicon multielectrode shank in the forepaw region of S1. The device had 16 evenly spaced microelectrodes that spanned all cortical layers (Figure 1A).

A stimulus response analysis (Figures 1B,C) and a post-mortem electrolytic lesion (Chen et al., 2009; Figures 1C,D) were used to confirm the implantation site and depth, respectively (see section “Materials and Methods”).

### Initial Sensitivity to Microstimulation

**FIGURE 2:**
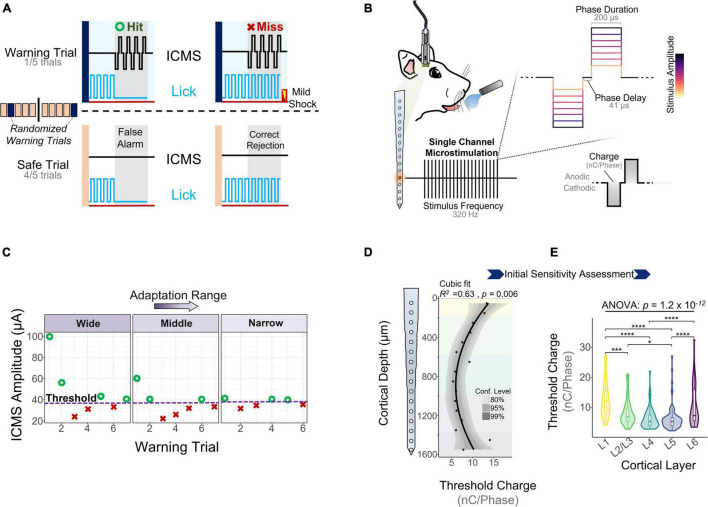
Behavioral paradigm and microstimulation sensitivity over depth. **(A)** A conditioned avoidance behavioral paradigm was used to assess the ability of the animals to detect microstimulation. **(B)** Stimulation frequency, phase duration, and phase delay were kept constant throughout the experiment. Stimulus amplitude was modulated based on the animal’s behavior using an adaptation protocol. **(C)** Adaptation protocol used to measure detection thresholds. Similar detection thresholds can be obtained regardless of the initial stimulation amplitudes. **(D)** Sensitivity of S1 to microstimulation as a function of cortical depth during the first five experimental sessions (*N* = 6). **(E)** Quantification of these thresholds across cortical layers (ANOVA with Tukey’s *post-hoc* test) (*N* = 6). *p ≤ 0.05, ***p ≤ 0.001, ****p ≤ 0.0001.

One key goal of the study was to assess how the sensitivity of S1 to ICMS changed across cortical depth and over time. To measure the ability of animals to detect ICMS we used a conditioned avoidance behavioral paradigm (Heffner and Heffner, 1995; Urdaneta et al., 2019, 2021; Saldanha et al., 2021). In this task, water-deprived rats were trained to stop licking from a spout upon presentation of an ICMS stimulus (Figure 2A, referred to as a “hit”). If the animal failed to stop drinking, the trial was considered a miss (Figure 2A, miss) and the animal received a mild electrocutaneous shock. Safe trials were used as a control to determine the animal’s licking behavior in the absence of an ICMS stimulus (Figure 2A, bottom). Stimulation parameters were held constant throughout the duration of the study except for amplitude (Figure 2B), which was modulated based on the animal’s response to determine ICMS detection thresholds (Figure 2C).

Experimental sessions started after 5 days of post-surgical recovery and consisted of obtaining detection thresholds from randomly selected channels until the animal was satiated. To determine the initial sensitivity of S1 to ICMS, we collected detection thresholds across all channels for 5 experimental sessions during the first 2 WPI. Figure 2D shows how the charge (amplitude × phase duration) necessary to evoke a response in S1 varies with cortical depth, with the most sensitive channels around 1 mm from the cortical surface (*R*^2^ = 0.63, *p* = 0.006, Cubic fit). Laminar quantification showed that detection thresholds change significantly across layers (Figure 2E). The mean charge necessary to elicit a threshold in L5 (6.37 ± 3.93 nC⋅Phase^–1^) and L4 (6.49 ± 3.47 nC⋅Phase^–1^) was roughly half that in L1 (12.91 ± 6.09 nC⋅Phase^–1^) and L6 (10.68 ± 6.66 nC⋅Phase^–1^) (Figure 2E). These depth-dependent microstimulation thresholds served as a foundation to investigate how the long-term stability of ICMS in S1 changes across cortical layers.

### Depth Dependent Longitudinal Microstimulation Performance

**FIGURE 3:**
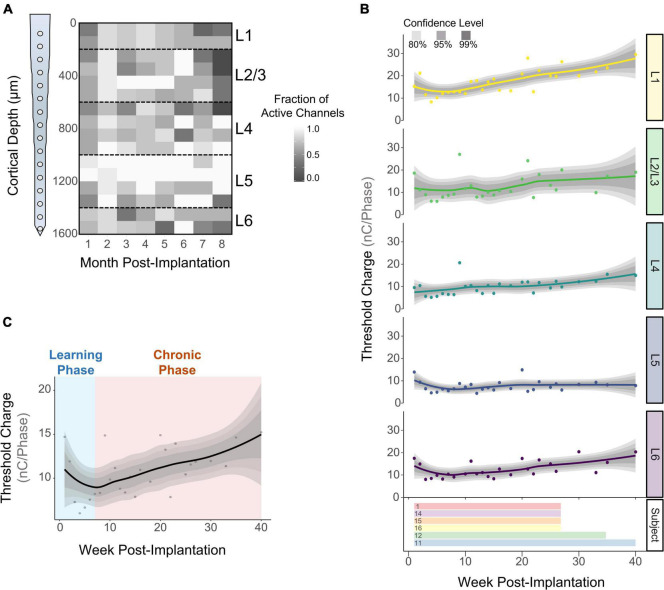
Long term stability of ICMS across cortical layers. The fraction of active channels was computed by assessing the number of times an electrode-site was able to elicit thresholds at safe ICMS charges. **(A)** Average fraction of active channels over time across subjects (*N* = 6). **(B)** Longitudinal detection thresholds across cortical layers (top) and the amount of time post-implantation in which detection thresholds were obtained from each subject (bottom). **(C)** Average detection thresholds of all cortical layers showing an initial decrease in thresholds (learning phase), followed by a period of increased thresholds (chronic phase) (*N* = 6). For **(B,C)**: lines represent a locally fitted polynomial regression of the means and shaded areas represent confidence intervals.

We measured detection thresholds across all channels up to 40 WPI by determining thresholds at least twice a week during the first 16 WPI, and at least twice a month afterward. If an electrode-site was able to elicit behavior at charges below 30 nC⋅Phase^–1^, it was deemed active (see section “Materials and Methods”). The fraction of active channels (FAC) was computed by dividing the number of times an electrode was active over the number of experimental sessions for each subject. Figure 3A shows the average FAC across subjects (*N* = 6) over time. The FAC across all electrode-sites peaked in the second month post-implantation (0.90 ± 0.08) with roughly twice the number of active channels compared to 8 months post-implantation (0.54 ± 0.3). This result suggests that the number of electrodes able to evoke thresholds changes over time. In addition, the average FAC across the duration of the study was at least 18.9% higher for L5 (0.87 ± 0.19) than for any other layer. To expand on these layer-dependent changes in chronic stability, we analyzed how the charge necessary to evoke a response changed over time across cortical layers. Figure 3B shows an initial decrease in detection thresholds across all layers until reaching a minimum around the 7th week post-implantation. This is followed by a steep increase in mean thresholds of 134 and 96% until the week 40 for L1 and L6, respectively. In contrast, L5 remained more stable with an increase in thresholds of only 42% (Figure 3B). The initial decrease in thresholds (Figure 3C, blue shade) has been reported in the literature as subjects improve on the behavioral task, regardless of the time post-implantation (Callier et al., 2015). Hence, we denominated this period the learning phase. Given the layer-specific differences at chronic timespoints following the learning phase, the period following the minimum threshold was designated the chronic phase (Figure 3C, orange shade). To further understand how thresholds change across subjects and across layers during these time periods, we examined the learning and chronic phases in the following sections.

### Learning Phase

**FIGURE 4:**
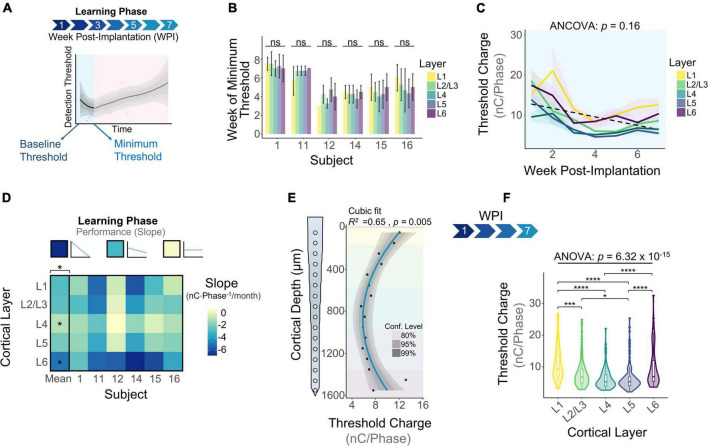
Microstimulation detection thresholds during the learning phase. **(A)** Graphical representation of the period of initial decrease in thresholds (learning phase) in the context of longitudinal thresholds. **(B)** Bar plot graph showing the week in which subjects reached minimum thresholds across cortical layers. **(C)** Average detection thresholds during the learning phase. Dashed line represents a linear regression of the mean. **(D)** Heatmap of the slope of thresholds between the baseline and week of minimum threshold across subjects. **(E)** Sensitivity of thresholds during the learning phase and **(F)** laminar quantification. Analysis of variance and pairwise comparisons between layers were performed with a one-way ANOVA with Tukey’s *post-hoc* test. Error bars in **(B)** and shaded areas in **(C)** indicate mean ± SEM. *p ≤ 0.05, ***p ≤ 0.001, ****p ≤ 0.0001.

The learning phase consisted of the period of initial decrease in detection thresholds observed on the first week of thresholds and up to the week in which thresholds reach a minimum (Figure 4A). Our first goal was to determine when the minimum threshold was reached for each subject. Figure 4B shows that on average, subjects reach the minimum threshold at different timespoints, ranging from 3.85 ± 0.72 weeks (subject 12) up to 7.25 ± 0.25 weeks (subject 1) post-implantation. We ran an analysis of variance to determine if the week of minimum threshold changed across layers for each animal, finding no statistical significance for any animals (Figure 4B: *p* > 0.05, One-way ANOVA). These observations suggest that animals reach the minimum threshold at different weeks, but this point is reached at roughly the same time across layers in each subject. Next, we plotted detection thresholds of the first 7 WPI across layers (Figure 4C). On average, detection thresholds decreased 44.1% from week 1 to week 7 at a rate of −1.93 nC⋅Phase^–1^/month (Figure 4C). The inclusion of time as a covariate revealed no statistically significant differences in thresholds across layers during the first 7 WPI (Figure 4C). We used the slope of thresholds between the first session and the week of minimum threshold for each animal across layers as a performance metric for ICMS stability. Figure 4D shows that the mean slope of thresholds during this time period changes significantly across layers (Figure 4D). On average, L4 (−0.97 ± 0.95 nC⋅Phase^–1^/month) had the shallowest slope, while L6 (−4.99 ± 1.47 nC⋅Phase^–1^/month) had the steepest slope (Figure 4D). These results suggest layer-specific differences in learning tactile behavior in S1, consistent with previous reports (Diamond et al., 1994). To assess if the overall depth-dependent sensitivity to ICMS shifted during the learning phase, we quantified cumulative thresholds during the first 7 WPI across cortical depth (Figures 4E,F). Comparable to the initial sensitivity assessment of the first five experimental sessions (Figures 2A,B), the overall ICMS sensitivity during the learning phase is non-uniform across depth (*R*^2^ = 0.65, *p* = 0.005, Cubic fit). L4 and L5 also persisted as the most sensitive layers (Figures 4E,F). Altogether, these observations indicate that the rate of threshold improvement as well as the overall sensitivity to ICMS during the learning phase change in a layer dependent manner.

### Chronic Phase

**FIGURE 5:**
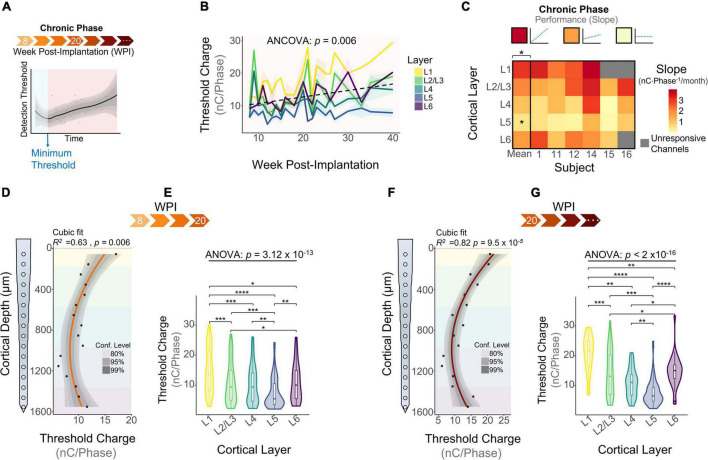
Microstimulation detection thresholds during the chronic phase. **(A)** Graphical representation of longitudinal threshold showing the beginning of the chronic phase (orange shade) after the week of minimum thresholds. **(B)** Average detection thresholds during the chronic phase. Dashed line represents a linear regression of the mean. **(C)** Heatmap of the slope of thresholds between the week of minimum threshold and the last week of threshold across subjects. ICMS Sensitivity across cortical depth and laminar quantification during **(D,E)** 8–20 weeks-post-implantation and **(F,G)** >20 WPI. Analysis of variance and pairwise comparisons between layers were performed with a one-way ANOVA with Tukey’s *post-hoc* test. Shaded areas in **(B)** indicate mean ± SEM. *p ≤ 0.05, **p ≤ 0.01, ***p ≤ 0.001, ****p ≤ 0.0001.

The chronic phase started immediately after the learning phase and lasted the remaining duration of the study (Figure 5A). This period of time is characterized by an overall increase in detection thresholds across all layers (Figure 5B: 0.79 nC⋅Phase^–1^/month, linear regression slope). We ran an ANCOVA to evaluate threshold differences across layers while controlling for the effects of time post-implantation. Our results showed significant differences in thresholds across cortical layers throughout the chronic phase (Figure 5B). To further elucidate the long-term stability of ICMS and account for different animal end points, the average slope per layer was calculated for each subject during the chronic phase. The heatmap in Figure 5C shows that the mean slope changes significantly across cortical layers. The cortical layer that showed the least steep slope over time was L5, increasing at an average rate of 0.65 ± 0.49 nC⋅Phase^–1^/month, indicating that L5 has the most stable thresholds over time. In contrast, L1 displays the sharpest increase in thresholds (3.25 ± 0.51 nC⋅Phase^–1^/month). For some subjects, channels in L1 and L6 were unresponsive to stimulation charges below 30 nC⋅Phase^–1^ by the endpoint (25 WPI) (Figure 5C, unresponsive channels). This result is consistent with our previous observation that the FAC across the duration of the study varied in a layer-dependent manner (Figure 2A). Lastly, to assess how ICMS sensitivity patterns change at chronic timespoints, we analyzed cumulative detection thresholds over depth at two different time periods in the chronic phase: from 8 to 20 WPI and >20 WPI. For both of these time points the ICMS sensitivity maintained a non-uniform trend across cortical depth (Figures 5D,E). Further laminar quantification revealed that sensitivity differences across cortical layers become more pronounced at chronic timespoints (Figures 5F,G). In particular, the percent increase in threshold amplitudes between L1 and L5 increased from 66.6% during 8–20 WPI to 91.0% on >20 WPI. These observations indicate that although the overall sensitivity pattern across cortical depth remains constant over time, threshold differences across layers become exacerbated in chronic time points. Altogether, these results reveal that the long-term stability of ICMS in S1 is dependent on the interfacing depth, raising the question of how biotic factors such as the FBR could be contributing to this depth-specific performance.

### The Extent of the Astrocytic Glial Scar Over Depth

**FIGURE 6:**
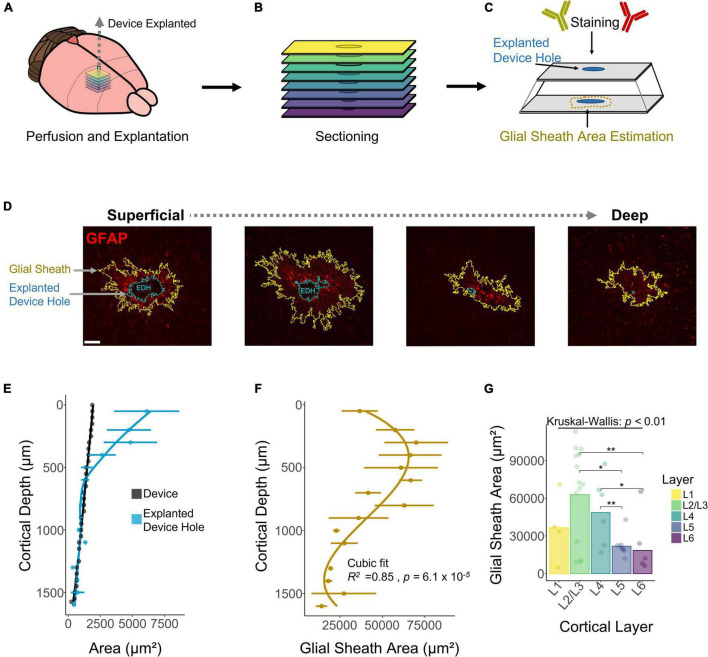
Histological assessment and quantification of the astrocytic glial scar across cortical depth. Histological analysis across cortical depth was performed using DeepHisto. **(A)** After perfusion, the device was carefully extracted. **(B)** Horizontal tissue slices surrounding the device were systematically sectioned to keep track of cortical depth. **(C)** Samples were stained with GFAP and the area of the glial scar was measured. **(D)** Representative histological sections across cortical depth for one subject. Yellow outlines represent the astrocytic glial scar surrounding the electrode and the blue outlines represent the explanted device hole. **(E)** Quantification of the area of the explanted device hole in comparison with the cross-sectional area of the device. Lines represent a locally fitted polynomial regression of the mean. **(F)** Area of the astrocytic glial scar as a function of cortical depth. Line represents a cubic fit. **(G)** Laminar quantification of the area of the astrocytic glial scar. Analysis of variance and pairwise comparisons between layers were performed with a Kruskal–Wallis test and a Wilcoxon rank-sum test with Holm adjustment, respectively. Error bars in **(E,F)** indicate mean ± SEM. *p ≤ 0.05, **p ≤ 0.01.

A decay in chronic performance of implantable microelectrodes has been associated with the FBR, specifically the astrocytic glial scar engulfing the electrode (Prasad and Sanchez, 2012; Prasad et al., 2014; Esquibel et al., 2020). The fluorescent intensity of glial fibrillary acidic protein (GFAP), a marker for astrocytes and indicative of the glial scar, has been reported to be higher near the cortical surface (McConnell et al., 2009). However, how the area of the astrocytic glial sheath varies across layers remains unexplored. To assess this question, we used DeepHisto (citation in review), a technique to evaluate the FBR across cortical depth. Following perfusion, systematic sectioning, and staining with GFAP (Figures 6A–C), the areas of the glial sheath and the explanted device hole (EDH) were measured using an automatic tracing tool (see section “Materials and Methods”). Figure 6D shows representative histological sections in which the areas of the EDH (blue outline) and the glial sheath (yellow outline) are non-uniform across cortical depth. Figure 6E shows how the average area of the EDH is up to 69.37% larger than the area of the device near the cortical surface. As we go down in cortical depth the area difference between the EDH and the device decreases, becoming almost negligible below 500 μm from the cortical surface. Given that both L1 and L6 had similar sensitivities to ICMS (Figure 2D), it is unlikely that this phenomenon was caused by higher microstimulation charges. Hence, it is more likely that this difference was caused by the device explantation or by micromotion.

Next, we quantified the area of the glial sheath engulfing the electrode. Figure 6F shows that the extent of the glial sheath is non-uniform across cortical depth (*R*^2^ = 0.85, *p* = 6.1 × 10^–5^), peaking at 300 μm (69954 ± 41861 μm^2^) from the cortical surface. Laminar quantification revealed that the area of the glial sheath is larger in L2/3 (Figure 6F) and changes significantly across cortical layers (Figure 6G). These observations reveal that the extent of astrocytic glial scar of chronically implanted electrodes can vary depending on the interfacing depth.

### The Microglial Response Over Depth

**FIGURE 7:**
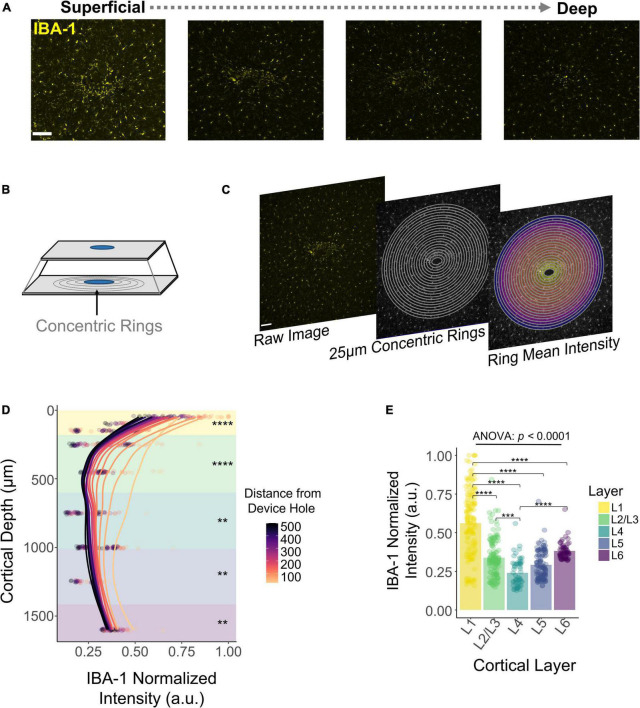
Histological response of microglia and macrophages across cortical depth. **(A)** Representative histological sections stained with Iba1 for one subject showing a change in fluorescence intensity across cortical depth. **(B)** Graphical representation of concentric rings around the explanted device hole. **(C)** Concentric rings and mean intensity calculation from a representative tissue section. **(D)** Quantification of Iba1 fluorescence intensity with respect to the distance from the device hole across cortical depth. Lines represent a locally fitted polynomial regression of the mean of each concentric ring. **(E)** Quantification across layers of the Iba1 fluorescence intensity. Analysis of variance and pairwise comparisons between layers were performed with a one-way ANOVA with Tukey’s *post-hoc* test. **p ≤ 0.01, ***p ≤ 0.001, ****p ≤ 0.0001.

DeepHisto sections were stained with Iba1, a marker specific for microglia/macrophages (Sasaki et al., 2001). Figure 7A shows that the fluorescent intensity of Iba1 is non-uniform across cortical depth, consistent with previous observations (Woolley et al., 2013). To quantify this pattern, we drew concentric rings diverging from the EDH (Figure 7B) and calculated the mean fluorescent intensity of each ring (Figure 7C). Similar to previous work (Biran et al., 2005; Gaire et al., 2018), our results show that fluorescent intensity of iba-1 consistently increased with proximity to the electrode (Figure 7D). Moreover, this change in fluorescent intensity became more pronounced in superficial layers (Figure 7E). Notably, the overall fluorescence intensity of microglia/macrophages for chronically implanted electrodes is higher near the cortical surface (*N* = 5). Laminar quantification showed a highly significant difference in Iba1 intensity across cortical layers [*F*_(4,531)_ = 90.19, *p* < 2 × 10^–16^; one-way ANOVA] with the highest intensity in L1 (Figure 7E; L1 vs. L2/3, L4, L5, L6 : *p* < 0.00001, Tukey’s *post-hoc* test). These findings indicate that the microglia/macrophage response to chronically implanted electrodes changes as a function of proximity to the electrode and as a function of cortical depth.

## Discussion

Our finding that longitudinal microstimulation had an initial decrease in thresholds followed by a period of irregular long-term stability complements previous ICMS work (Rousche and Normann, 1998; Bartlett et al., 2005; Koivuniemi et al., 2011; Callier et al., 2015; Hughes et al., 2021) while revealing layer-specific responses (Urdaneta et al., 2021). Consistent with our findings, several studies have reported an initial decrease in thresholds followed by a period of increased (Rousche and Normann, 1998; Koivuniemi et al., 2011) or stable thresholds (Bartlett et al., 2005; Callier et al., 2015; Hughes et al., 2021). However, the duration of the initial decrease in thresholds (learning phase) varies across the literature. Studies have reported a decrease in thresholds that reaches a minimum after days (Rousche and Normann, 1998; Bartlett et al., 2005; Koivuniemi et al., 2011), weeks (Rousche and Normann, 1998), and months after implantation (Callier et al., 2015; Hughes et al., 2021). Interestingly, this period of ICMS threshold improvement is even present in animals for which the ICMS regime started years after implantation (Callier et al., 2015). This suggests that the decrease in ICMS thresholds is not necessarily related to acute biological changes occurring after implantation (Biran et al., 2005; Kozai et al., 2012; Salatino et al., 2017). Instead, this decrease could be related to the subject’s ability to detect and interpret ICMS in the context of the behavioral task. In addition, our observation that the rate of change of threshold amplitudes varied significantly across layers could be related to layer-specific differences in cortical adaptation. Notably, L4 had the shallowest decrease in thresholds during the learning phase, and neurons in this layer have been shown to have the lowest levels of plasticity in tactile learning in adult rats (Diamond et al., 1994).

Following the learning phase, our average ICMS threshold charge increased during the chronic phase in a layer-dependent manner. Previous studies have found different chronic stability responses to ICMS, with some reporting increases in thresholds (Bartlett et al., 2005; Koivuniemi et al., 2011; Davis et al., 2012) and others reporting stable thresholds (Callier et al., 2015; Hughes et al., 2021). We found that whether thresholds increased or remained stable was highly dependent on the cortical layer. This finding could provide insights into the interelectrode variability observed in previous studies (Rousche and Normann, 1998; Hughes et al., 2021). For instance, Hughes et al. (2021) reported that the long-term stability of ICMS in humans implanted with a Utah array in S1 varied across electrodes, and that electrodes with high thresholds were clustered together^13^. It is possible that, due to the non-planar geometry of the human cortex, some of the electrodes rested at a different cortical depth, leading to different ICMS stability responses over time. Based on our observation that the ICMS sensitivity of S1 changes across depth, it is possible that some of these electrodes were located at a different cortical depth, producing distinct long-term stability responses.

Our DeepHisto analysis showed that the overall fluorescent intensity of microglia/macrophages was higher in L1, consistent with previous observations. Different groups have reported that the extent of the microglial response is greater near the cortical surface at 4 WPI (Woolley et al., 2013) and up to 16 WPI (McConnell et al., 2009) for chronically implanted rats. Our findings expand on these studies by showing that this depth-dependent microglial trend persists up to 40 WPI. In the healthy brain, microglia play an important role in maintaining neuronal signaling and synapse regulation (Tremblay et al., 2011). However, microglia become activated after implantation, causing a change in morphology and the release of proinflammatory cytokines and reactive oxygen species (Polikov et al., 2005). Hence, long-term activation of microglia in a depth-dependent manner can have important implications for the health and function of cells in superficial cortical layers.

In addition, our assessment of the astrocytic glial scar engulfing the electrode revealed that its extent is also non-uniform across cortical depth, peaking around L2/3. Previous work has reported qualitative differences in FBR with increased responses near the cortical surface (Kozai et al., 2014; Nolta et al., 2015). McConnell et al. (2009) assessed the GFAP response of chronically implanted rats across four representative cortical depths (200, 600, 1000, and 1400 μm); these rats were implanted for different time periods ranging from 2 to 16 WPI (McConnell et al., 2009). The fluorescent intensity levels of GFAP decreased as a function of cortical depth after 8 WPI. These depth-dependent results were partially attributed to the thickness of the device, which tapers down as a function of cortical depth (McCreery et al., 1990). Our electrodes had a similar geometry; however, the increased spatial resolution of our assessment revealed that the extent of the glial scar is non-monotonic across depth. Specifically, we found that the area of the glial sheath did not peak at the depth of maximum thickness of the device (cortical surface and L1), instead reaching its maximum at 300 μm (L2/3). These layer-dependent changes in the glial scar could arise from morphological differences in astrocytes across cortical depth. Indeed, both the territorial volume of astrocytes as well as the orientation angle relative to the cortical surface peak at L2/3 (Lanjakornsiripan et al., 2018). Furthermore, the molecular profile of astrocytes also varies across layers (Bayraktar et al., 2020). For instance, the expression of *Chrdl1* in astrocytes peaks in L2/3 for both mice and humans (Bayraktar et al., 2020). *Chrdl1* is a bone morphogenetic protein (BMP) inhibitor (Cyr-Depauw et al., 2016), a key molecule in the regulation of the glial scar (Zhong and Zou, 2014). Further research is necessary to investigate the relationship between electrode size/geometry and the extent of the glial sheath across cortical depth. In addition, understanding the complexity of the glial scar across cortical depth can better inform groups developing therapeutics such as drug-delivery and coatings (Sommakia et al., 2014) for the mitigation of the FBR.

Changes in neuronal morphology and the FBR might lead to differences in ICMS threshold stability. Consistent with previous reports (Koivuniemi et al., 2011), our results showed that microstimulation electrodes near the cortical surface had lower long-term stability. The fact that the glial scar and microglia/macrophage densities are exacerbated in superficial layers could lead to the presumption that the FBR is directly responsible for the decay in ICMS chronic performance. However, this degradation process is more complex. The fluorescent intensity of microglia/macrophages was lower at deeper layers and the area of the glial sheath was the lowest in L6. Concurrently, this layer had one of the poorest ICMS longitudinal performances. Hence, the layer specific decay in longitudinal stability of ICMS could not be solely attributed to depth-specific changes in the FBR. The chronic stability of implantable microelectrodes is a multivariate process constituted by abiotic and biotic factors. For instance, abiotic factors such as the structural integrity of the device, composition of the electrode material (Prasad et al., 2014) (i.e., flexibility) and its dimensions represent abiotic factors that might affect the long-term stability of ICMS. Analogously, biotic factors such as the health and function of neurons surrounding the electrode (Biran et al., 2005; Salatino et al., 2017) can also affect the behavioral response to ICMS. Indeed, layer-specific changes in neuronal morphology and function (Lanjakornsiripan et al., 2018; Gouwens et al., 2019) could lead to different sensitivities to ICMS (Aberra et al., 2018; see: Urdaneta et al., 2021) for an extended discussion). We found that channels with high initial thresholds tended to have fewer active channels over time with higher detection thresholds over time. Future research is necessary to assess the impact of chronic microstimulation charges in the health and function of neuronal populations across cortical layers.

In conclusion, we found that the long-term sensitivity to ICMS and the number of active sites over time is layer dependent. Similarly, a post-mortem analysis showed that the astrocytic glial scar and microglia response are non-uniform across cortical depth. This approach to evaluating the long-term ICMS performance in S1 and the FBR across cortical depth have important implications in the design of neuroprosthetic devices and future ICMS studies.

## Materials and Methods

### Device Implantation

All animal experiments and surgeries were performed under the approval and guidance of the University of Florida’s Institutional Animal Care and Use Committee. The specific details of the surgical implantation have been described elsewhere. Briefly, six male Sprague-Dawley rats (450–650 g, Charles River, Chicago, IL, United States) were initially induced with 5% isoflurane (Zoetis, Parsippany, NJ, United States) in oxygen at 1.5–2 L/min. The isoflurane was reduced after 5 min and sustained at 1.5–3% throughout the surgical procedure. Meloxicam (1–2 mg/kg, SQ, Loxicom, Norbrook Laboratories, Newry, Northern Ireland) was administered subcutaneously. A 1 mm^2^ cranial window was performed over the forepaw region of the primary somatosensory cortex [0.5 mm, 3.5 mm (Paxinos and Watson, 2006)]. Burr holes were drilled to secure four titanium bone screws (United Titanium, OH, United States). After a durotomy, a micro-insertion system (PiLine M663, Physik Instrumente, Karlsruhe, Germany) was used to implant a silicon microelectrode device (A1 × 16–3 mm-100-703-HZ16, NeuroNexus, Ann Arbor, MI, United States). The device (previously sterilized with ethylene oxide) was rinsed with sterile saline and inserted 1.6 mm from the cortical surface at 100 mm/s. This device had 16 iridium oxide electrode-sites with a surface area of 703 μm^2^ with a pitch of 100 μm (Figure 1A). The site of the craniotomy was filled with a silicone elastomer (Kwik-Sil, WPI, Sarasota, FL, United States). Once the elastomer thickened, UV-cured dental composite (DentalSource, CA, United States) was used to secure the connectors in place. After surgical recovery, the animal was placed in a Faraday cage and an air puff stimulus was directed to the contralateral forepaw while neural recordings were obtained using a PZ5 Neuro digitizer amplifier (Tucker Davis Technologies, Alachua, FL, United States). The custom-made air puff system was controlled by an RZ5D Bioamp processor (Tucker Davis Technologies, Alachua, FL, United States). Epochs from the air puff were used to calculate an inverse current source density (iCSD) (Pettersen et al., 2006) using MATLAB (2019b, Mathworks, MA, United States). Moreover, coronal sections stained for Anti-VGluT2 Antibody (1:500–AB2241, Sigma-Aldrich) were used to corroborate the length of cortical layers in S1 (Meyer et al., 2013).

### Behavioral Paradigm

A conditioned avoidance behavioral paradigm was used to assess the ability of rats to detect ICMS. Specific details have been described elsewhere (Koivuniemi et al., 2011). The animal’s behavior was monitored through a custom-made RPvds code and a RZ5D Bioamp processor (Tucker Davis Technologies, Alachua, FL, United States). Briefly, rats were trained to stop licking behavior upon presentation of an ICMS stimulus (Figure 2A). If the animal failed to stop drinking, a percutaneous shock was delivered through the spout. Trials were organized into blocks consisting of one warning trial and four safe trials. The ICMS stimulus was only present in warning trials and the order of the warning trial within a block was pseudo-randomly chosen. Safe trials contained no stimulus and were used to keep track of the licking behavior and ignore trials in which the animal stopped drinking in the absence of stimulus. Specifically, a false alarm was counted if the animal stopped drinking for more than 20% out of the 650 ms decision window. If a block of five trials contained more than two false alarms, the entire block was invalidated and repeated. Analogously, if during a warning trial the animal successfully avoided licking for more than 20% of the ICMS stimulus presentation phase (650 ms), the trial was considered a hit; otherwise, it was considered a miss (Figure 2A). If an animal successfully avoided to lick (hit), the stimulus amplitude of the next warning trial was reduced. Conversely, if the animal failed the trial, the stimulus amplitude was increased. A shift from hit to miss and vice versa was denominated a reversal. After three reversals, the average of amplitude of the last five trials was calculated and determined as the detection threshold.

### Longitudinal Microstimulation Experiments

Microstimulation was delivered to a single electrode-site on the implanted device *via* an IZ-32 stimulator with an LZ48-200 battery (Tucker-Davis Technologies, Alachua, FL, United States). Microstimulation consisted of 0.2 ms phase duration cathode-leading symmetric waveforms with a phase delay of 0.04 ms and a frequency of 320 Hz. Detection thresholds were reported in charge per phase (nC) by multiplying the stimulus amplitude (μA) by the phase duration (ms). Experimental sessions started after 5 days of post-surgical recovery. Each session consisted of obtaining detection thresholds from randomly selected channels (typically 8 to 16 channels) until the animal was satiated. A custom-made metallic protective cap was placed on the headstage after each session to avoid damage to the connectors. Thresholds were measured at least twice a week during the first 16 WPI, and at least twice a month afterward. To avoid tissue damage due to high microstimulation charges (Cogan et al., 2016), the total charge delivered was limited to a maximum of 30 nC⋅Phase^–1^ (Urdaneta et al., 2021).

### Tissue Processing and Cryosectioning

After conclusion of the ICMS experiments, subjects were anesthetized with 5% isoflurane in 2 L/min oxygen. A transcardial perfusion was carried out using phosphate buffered saline (PBS) followed by 4% paraformaldehyde (PFA) solution. Next, the skulls were incubated in 4% PFA at 4°C for 3 days. After several washes in PBS, the skulls were surgically resected, and the microelectrode was meticulously pulled out of the cortex (Figure 6A). The brains were then incubated in a 30% sucrose solution at 4°C for cryoprotection. Once the samples sank into the bottom of the vial (typically after 72 h), these were flash frozen in 2-methylbutane (Sigma Aldrich, St. Louis, MO, United States) at −40°C. Next, tissue within a 5 mm radius from the EDH was resected and mounted on a cryosection chuck with Tissue-Plus O.C.T. Compound (23-730-571, Fisher). The samples were then serially sectioned to track cortical depth. In brief, 20 μm tissue sections tangential to the explanted device were sliced using a cryostat (CM 1520, Leica) at −20°C. The first tissue slice on slide 1 represents a cortical depth of 0 to 20 μm, the first slice on slide 2 corresponds to cortical depth 20 to 40 μm, and so on (Figure 6B).

### Immunohistochemistry and Imaging

After leaving the slides at room temperature for 1 h, they were washed with PBS in three 5 min cycles. Next, the slides were incubated in Animal-free R.T.U. Blocker (SP-5035, Vector Laboratories Inc.) overnight at 4°C. To identify astrocytes and microglia/macrophages, immunohistochemistry was performed using the primary antibodies Anti-GFAP (CPCA-GFAP, EnCor Biotech.) and Anti-Iba-1 (019-19741, Wako Chem.), respectively. Primary antibodies were diluted to 1:500 in Animal-free Blocker, R.T.U (SP-5035, Vector Laboratories Inc). The primary antibodies were then distributed across the slides and incubated at 4°C for 36 h (Figure 6C). Following three 10 min PBS wash cycles, slides were stained with secondary antibodies (Cy3 anti-rabbit, 711-165-152, Jackson Immuno.) (Cy5 anti-chicken, 703-175-155, Jackson Immuno.) diluted 1:500 in animal-free blocker and incubated at 4°C for 24 h. After three 10 min PBS washes the excess liquid was aspirated, and glass coverslips were mounted using Vector Shield medium with DAPI (H-1200, Vector Labs). Slides were kept in the dark at RT for 24 h before imaging. Image acquisition was performed on a Leica DMi8 microscope running LAS X Premium software. Peripheral components include a DFC9000 GT camera, EL-6000 light source (30 ms), HC PL APO 10x/0.45 objective, and Leica Y3 and Y5 filter cubes. Acquisitions were centered over the EDH (Figure 6C).

### Foreign Body Response Quantification

After manual selection of the area of the EDH, the mean gray value intensity of 25 μm concentric rings around the device was measured. This process was repeated across cortical depth. The fluorescence intensity values were then normalized using min-max normalization and plotted across cortical depth. Quantification of the glial scar area was traced with an automatic tracing tool in which the tolerance value was manually adjusted by blind evaluators. All slide quantification analyses were performed with a custom-made script in Fiji (Schindelin et al., 2012).

### Statistics

Parametric comparisons were assessed *via* one-way ANOVA followed by a Tukey’s *post-hoc* test. Layer-specific differences in thresholds with time as a covariate were assessed using an ANCOVA. For datasets with non-normal distribution (Shapiro Wilks test) or with less than 20 samples per group (Figure 7), analysis of variance and pairwise comparison were performed *via* a Kruskal–Wallis test and a Wilcoxon rank-sum test with Holm adjustment, respectively. *p ≤ 0.05, ^**^p ≤ 0.01, ^***^p ≤ 0.001, ^****^p ≤ 0.0001. All statistical analyses were performed in R Statistical Software Version 4.0.0 (Vienna, Austria).

## Data Availability Statement

The raw data supporting the conclusions of this article will be made available by the authors, upon reasonable request.

## Ethics Statement

The animal study was reviewed and approved by the University of Florida Institutional Animal Care and Use Committee (IACUC).

## Author Contributions

MU, NK, SC, FD, SF, and KO conceived and designed the study and reviewed and edited the manuscript. MU, NK, and FD set up the behavioral rig. MU performed the surgical implantations and wrote the manuscript. MU and NK collected threshold data and analyzed the microstimulation and histological data. SC performed histology and imaging. All authors contributed to the article and approved the submitted version.

## Conflict of Interest

The authors declare that the research was conducted in the absence of any commercial or financial relationships that could be construed as a potential conflict of interest.

## Publisher’s Note

All claims expressed in this article are solely those of the authors and do not necessarily represent those of their affiliated organizations, or those of the publisher, the editors and the reviewers. Any product that may be evaluated in this article, or claim that may be made by its manufacturer, is not guaranteed or endorsed by the publisher.

## References

[B1] Aberra A. S. Peterchev A. V. Grill W. M. (2018). Biophysically realistic neuron models for simulation of cortical stimulation. *J. Neural Eng.* 15:066023. 10.1088/1741-2552/aadbb1 30127100 PMC6239949

[B2] Barrese J. C. Rao N. Paroo K. Triebwasser C. Vargas-Irwin C. Franquemont L. (2013). Failure mode analysis of silicon-based intracortical microelectrode arrays in non-human primates. *J. Neural Eng.* 10:066014. 10.1088/1741-2560/10/6/066014 24216311 PMC4868924

[B3] Bartlett J. R. DeYoe E. A. Doty R. W. Lee B. B. Lewine J. D. Negrão N. (2005). Psychophysics of electrical stimulation of striate cortex in macaques. *J. Neurophysiol.* 94 3430–3442. 10.1152/jn.00406.2005 16079195

[B4] Bayraktar O. A. Bartels T. Holmqvist S. Kleshchevnikov V. Martirosyan A. Polioudakis D. (2020). Astrocyte layers in the mammalian cerebral cortex revealed by a single-cell in situ transcriptomic map. *Nat. Neurosci.* 23 500–509. 10.1038/s41593-020-0602-1 32203496 PMC7116562

[B5] Biran R. Martin D. C. Tresco P. A. (2005). Neuronal cell loss accompanies the brain tissue response to chronically implanted silicon microelectrode arrays. *Exp. Neurol.* 195 115–126. 10.1016/j.expneurol.2005.04.020 16045910

[B6] Callier T. Schluter E. W. Tabot G. A. Miller L. E. Tenore F. V. Bensmaia S. J. (2015). Long-term stability of sensitivity to intracortical microstimulation of somatosensory cortex. *J. Neural Eng.* 12:056010. 10.1088/1741-2560/12/5/056010 26291448

[B7] Campbell A. Wu C. (2018). Chronically implanted intracranial electrodes: tissue reaction and electrical changes. *Micromachines* 9:430. 10.3390/mi9090430 30424363 PMC6187588

[B8] Chen Y. Y. Lai H. Y. Lin S. H. Cho C. W. Chao W. H. Liao C. H. (2009). Design and fabrication of a polyimide-based microelectrode array: application in neural recording and repeatable electrolytic lesion in rat brain. *J. Neurosci. Methods* 182 6–16. 10.1016/j.jneumeth.2009.05.010 19467262

[B9] Cogan S. F. Ludwig K. A. Welle C. G. Takmakov P. (2016). Tissue damage thresholds during therapeutic electrical stimulation. *J. Neural Eng.* 13:021001. 10.1088/1741-2560/13/2/021001 26792176 PMC5386002

[B10] Cyr-Depauw C. Northey J. J. Tabariès S. Annis M. G. Dong Z. Cory S. (2016). Chordin-like 1 suppresses bone morphogenetic protein 4-induced breast cancer cell migration and invasion. *Mol. Cell Biol.* 36 1509–1525. 10.1128/MCB.00600-15 26976638 PMC4859683

[B11] Davis T. S. Parker R. A. House P. A. Bagley E. Wendelken S. Normann R. A. (2012). Spatial and temporal characteristics of V1 microstimulation during chronic implantation of a microelectrode array in a behaving macaque. *J. Neural Eng.* 9:065003. 10.1088/1741-2560/9/6/065003 23186948 PMC3521049

[B12] DeYoe E. A. Lewine J. D. Doty R. W. (2005). Laminar variation in threshold for detection of electrical excitation of striate cortex by macaques. *J. Neurophysiol.* 94 3443–3450. 10.1152/jn.00407.2005 16079194

[B13] Diamond M. E. Huang W. Ebner F. F. (1994). Laminar comparison of somatosensory cortical plasticity. *Science* 265 1885–1888. 8091215 10.1126/science.8091215

[B14] Eles J. R. Vazquez A. L. Snyder N. R. Lagenaur C. Murphy M. C. Kozai T. D. (2017). Neuroadhesive L1 coating attenuates acute microglial attachment to neural electrodes as revealed by live two-photon microscopy. *Biomaterials* 113 279–292. 10.1016/j.biomaterials.2016.10.054 27837661 PMC5563378

[B15] Esquibel C. R. Wendt K. D. Lee H. C. Gaire J. Shoffstall A. Urdaneta M. E. (2020). Second harmonic generation imaging of collagen in chronically implantable electrodes in brain tissue. *Front. Neurosci.* 14:95. 10.3389/fnins.2020.00095 32733179 PMC7358524

[B16] Flesher S. N. Collinger J. L. Foldes S. T. Weiss J. M. Downey J. E. Tyler-Kabara E. C. (2016). Intracortical microstimulation of human somatosensory cortex. *Sci. Transl. Med.* 8 1–11. 10.1126/scitranslmed.aaf8083 27738096

[B17] Gaire J. Lee H. C. Hilborn N. Ward R. Regan M. Otto K. J. (2018). The role of inflammation on the functionality of intracortical microelectrodes. *J. Neural Eng.* 15:066027. 10.1088/1741-2552/aae4b6 30260321

[B18] Giulian D. Li J. Li X. George J. Rutecki P. A. (1994). The impact of microglia-derived cytokines upon gliosis in the CNS. *Dev. Neurosci.* 16 128–136. 10.1159/000112099 7535679

[B19] Gouwens N. W. Sorensen S. A. Berg J. Lee C. Jarsky T. Ting J. (2019). Classification of electrophysiological and morphological neuron types in the mouse visual cortex. *Nat. Neurosci.* 22 1182–1195. 10.1038/s41593-019-0417-0 31209381 PMC8078853

[B20] Harris K. D. Shepherd G. M. G. (2015). The neocortical circuit: themes and variations. *Nat. Neurosci.* 18 170–181. 10.1038/nn.3917 25622573 PMC4889215

[B21] Heffner H. E. Heffner R. S. (1995). “Conditioned avoidance,” in *Methods In Comparative Psychoacoustics*, eds Klump G. M. Dooling R. J. Fay R. R. Stebbins W. C. (Cham: Springer), 79–93.

[B22] Hughes C. L. Flesher S. N. Weiss J. M. Downey J. E. Boninger M. Collinger J. L. (2021). Neural stimulation and recording performance in human sensorimotor cortex over 1500 days. *J. Neural Eng.* 18:045012. 10.1088/1741-2552/ac18ad 34320481 PMC8500669

[B23] Koivuniemi A. Wilks S. J. Woolley A. J. Otto K. J. (2011). Multimodal, longitudinal assessment of intracortical microstimulation. *Prog. Brain Res.* 194 131–144. 10.1016/B978-0-444-53815-4.00011-X 21867800 PMC8098704

[B24] Kozai T. D. Y. Li X. Bodily L. M. Caparosa E. M. Zenonos G. A. Carlisle D. L. (2014). Effects of caspase-1 knockout on chronic neural recording quality and longevity: insight into cellular and molecular mechanisms of the reactive tissue response. *Biomaterials* 35 9620–9634. 10.1016/j.biomaterials.2014.08.006 25176060 PMC4174599

[B25] Kozai T. D. Y. Vazquez A. L. Weaver C. L. Kim S. G. Cui X. T. (2012). In vivo two-photon microscopy reveals immediate microglial reaction to implantation of microelectrode through extension of processes. *J. Neural Eng.* 9:66001. 10.1088/1741-2560/9/6/066001 PMC3511663 23075490

[B26] Lanjakornsiripan D. Pior B. J. Kawaguchi D. Furutachi S. Tahara T. Katsuyama Y. (2018). Layer-specific morphological and molecular differences in neocortical astrocytes and their dependence on neuronal layers. *Nat. Commun.* 9:1623. 10.1038/s41467-018-03940-3 29691400 PMC5915416

[B27] McConnell G. C. Rees H. D. Levey A. I. Gutekunst C. A. Gross R. E. Bellamkonda R. V. (2009). Implanted neural electrodes cause chronic, local inflammation that is correlated with local neurodegeneration. *J. Neural Eng.* 6:056003. 10.1088/1741-2560/6/5/056003 19700815

[B28] McCreery D. B. Agnew W. F. Yuen T. G. H. Bullara L. (1990). Charge density and charge per phase as cofactors in neural injury induced by electrical stimulation. *IEEE Trans. Biomed. Eng.* 37 996–1001. 10.1109/10.102812 2249872

[B29] Mercanzini A. Colin P. Bensadoun J. C. Bertsch A. Renaud P. (2009). In vivo electrical impedance spectroscopy of tissue reaction to microelectrode arrays. *IEEE Trans. Biomed. Eng.* 56 1909–1918. 10.1109/TBME.2009.2018457 19362904

[B30] Meyer H. S. Egger R. Guest J. M. Foerster R. Reissl S. Oberlaender M. (2013). Cellular organization of cortical barrel columns is whisker-specific. *Proc. Natl. Acad. Sci. U.S.A.* 110 19113–19118. 10.1073/pnas.1312691110 24101458 PMC3839692

[B31] Nolta N. F. Christensen M. B. Crane P. D. Skousen J. L. Tresco P. A. (2015). BBB leakage, astrogliosis, and tissue loss correlate with silicon microelectrode. *Biomaterials* 53 753–762. 10.1016/j.biomaterials.2015.02.081 25890770

[B32] Paxinos G. Watson C. (2006). *The rat Brain in Stereotaxic Coordinates: Hard Cover Edition.* Amsterdam: Elsevier.

[B33] Pettersen K. H. Devor A. Ulbert I. Dale A. M. Einevoll G. T. (2006). Current-source density estimation based on inversion of electrostatic forward solution: effects of finite extent of neuronal activity and conductivity discontinuities. *J. Neurosci. Methods* 154 116–133. 10.1016/j.jneumeth.2005.12.005 16436298

[B34] Polikov V. S. Tresco P. A. Reichert W. M. (2005). Response of brain tissue to chronically implanted neural electrodes. *J. Neurosci. Methods* 148 1–18. 10.1016/j.jneumeth.2005.08.015 16198003

[B35] Prasad A. Sanchez J. C. (2012). Quantifying long-term microelectrode array functionality using chronic in vivo impedance testing. *J. Neural Eng.* 9:026028. 10.1088/1741-2560/9/2/026028 22442134

[B36] Prasad A. Xue Q. S. Dieme R. Sankar V. Mayrand R. C. Nishida T. (2014). Abiotic-biotic characterization of Pt/Ir microelectrode arrays in chronic implants. *Front. Neuroengineering.* 7:2. 10.3389/fneng.2014.00002 24550823 PMC3912984

[B37] Romo R. Hernández A. Zainos A. Salinas E. (1998). Somatosensory discrimination based on cortical microstimulation. *Nature* 392 387–390. 10.1038/32891 9537321

[B38] Rousche P. J. Normann R. A. (1998). Chronic recording capability of the utah intracortical electrode array in cat sensory cortex. *J. Neurosci. Methods* 82 1–15. 10.1016/s0165-0270(98)00031-4 10223510

[B39] Rousche P. J. Normann R. A. (1999). Chronic intracortical microstimulation (ICMS) of cat sensory cortex using the Utah Intracortical Electrode Array. *IEEE Trans. Rehabil. Eng.* 7 56–68. 10.1109/86.750552 10188608

[B40] Salas M. A. Bashford L. Kellis S. Jafari M. Jo H. Kramer D. (2018). Proprioceptive and cutaneous sensations in humans elicited by intracortical microstimulation. *eLife* 7:e32904. 10.7554/eLife.32904 29633714 PMC5896877

[B41] Salatino J. W. Ludwig K. A. Kozai T. D. Y. Purcell E. K. (2017). Glial responses to implanted electrodes in the brain. *Nat. Biomed. Eng.* 1 862–877. 30505625 10.1038/s41551-017-0154-1 PMC6261524

[B42] Saldanha R. L. Urdaneta M. E. Otto K. J. (2021). The role of electrode-site placement in the long-term stability of intracortical microstimulation. *Front. Neurosci.* 15:712578. 10.3389/fnins.2021.712578 34566563 PMC8455844

[B43] Sasaki Y. Ohsawa K. Kanazawa H. Kohsaka S. Imai Y. (2001). Iba1 is an actin-cross-linking protein in macrophages/microglia. *Biochem. Biophys. Res. Commun.* 286 292–297. 10.1006/bbrc.2001.5388 11500035

[B44] Schindelin J. Arganda-Carreras I. Frise E. Kaynig V. Longair M. Pietzsch T. (2012). Fiji: an open-source platform for biological-image analysis. *Nat. Methods* 9 676–682. 10.1038/nmeth.2019 22743772 PMC3855844

[B45] Sommakia S. Lee H. C. Gaire J. Otto K. J. (2014). Materials approaches for modulating neural tissue responses to implanted microelectrodes through mechanical and biochemical means. *Curr. Opin. Solid State Mater. Sci.* 18 319–328. 10.1016/j.cossms.2014.07.005 25530703 PMC4267064

[B46] Tehovnik E. J. Slocum W. M. (2009). Depth-dependent detection of microampere currents delivered to monkey V1. *Eur. J. Neurosci.* 29 1477–1489. 10.1111/j.1460-9568.2009.06695.x 19519630 PMC3064858

[B47] Tremblay M. È Stevens B. Sierra A. Wake H. Bessis A. Nimmerjahn A. (2011). The role of microglia in the healthy brain. *J. Neurosci.* 31 16064–16069. 22072657 10.1523/JNEUROSCI.4158-11.2011 PMC6633221

[B48] Urdaneta M. E. Koivuniemi A. S. Otto K. J. (2017). Central nervous system microstimulation: towards selective micro-neuromodulation. *Curr. Opin. Biomed. Eng.* 4 65–77.

[B49] Urdaneta M. E. Kunigk N. G. Delgado F. Fried S. I. Otto K. J. (2021). Layer-specific parameters of intracortical microstimulation of the somatosensory cortex. *J. Neural Eng.* 18:055007. 10.1088/1741-2552/abedde 33706301 PMC12949851

[B50] Urdaneta M. E. Kunigk N. Delgado F. Otto K. J. Member S. (2019). “Somatosensory cortex microstimulation: behavioral effects of phase duration and asymmetric waveforms,” in *Proceedings of the 41st Annual International Engineering Medicine Biology Society*, (Berlin). 10.1109/EMBC.2019.8856579 31946248

[B51] Woolley A. J. Desai H. A. Otto K. J. (2013). Chronic intracortical microelectrode arrays induce non-uniform, depth-related tissue responses Related content. *J. Neural Eng.* 10:026007. 10.1088/1741-2560/10/2/026007 23428842 PMC4096286

[B52] Zhong J. Zou H. (2014). BMP signaling in axon regeneration. *Curr. Opin. Neurobiol.* 27 127–134. 10.1016/j.conb.2014.03.009 24713578 PMC4122622

